# 3-Benzyl-4-ethyl-1*H*-1,2,4-triazole-5(4*H*)-thione

**DOI:** 10.1107/S1600536812051276

**Published:** 2013-01-04

**Authors:** Zbigniew Karczmarzyk, Monika Pitucha, Waldemar Wysocki, Anna Pachuta-Stec, Andrzej Stańczuk

**Affiliations:** a Department of Chemistry, Siedlce University, ul. 3 Maja 54, 08-110 Siedlce, Poland; b Department of Organic Chemistry, Faculty of Pharmacy with Division of Medical Analytics, Medical University, ul. Chodźki 4A, 20-093 Lublin, Poland

## Abstract

The title compound, C_11_H_13_N_3_S, exists in the 5-thioxo tautomeric form. The benzene ring exhibits disorder with a refined ratio of 0.77 (2):0.23 (2) for components *A* and *B* with a common bridgehead C atom. The 1,2,4-triazole ring is essentially planar, with a maximum deviation of 0.002 (3) Å for the benzyl-substituted C atom, and forms dihedral angles of 88.94 (18) and 86.56 (49)° with the benzene rings of components *A* and *B*, respectively. The angle between the plane of the ethyl chain and the mean plane of 1,2,4-triazole ring is 88.55 (15)° and this conformation is stabilized by an intra­molecular C—H⋯S contact. In the crystal, pairs of N—H⋯S hydrogen bonds link mol­ecules into inversion dimers. π–π inter­actions are observed between the triazole and benzene rings, with centroid–centroid separations of 3.547 (4) and 3.544 (12) Å for components *A* and *B*, and slippages of 0.49 (6) and 0.58 (15) Å, respectively.

## Related literature

For background information on 1,2,4-triazole-5-thio­nes, see: Saadeh *et al.* (2010 ▶); Akhtar *et al.* (2008 ▶); Al-Omar *et al.* (2010 ▶). For their biological activity, see: Pitucha *et al.* (2010 ▶). For the synthesis, see: Dobosz & Pachuta-Stec (1996 ▶). For related structures, see: Karczmarzyk *et al.* (2012 ▶); Kruszynski *et al.* (2007 ▶); Siwek *et al.* (2008 ▶). For graph-set motifs, see Bernstein *et al.* (1995 ▶).
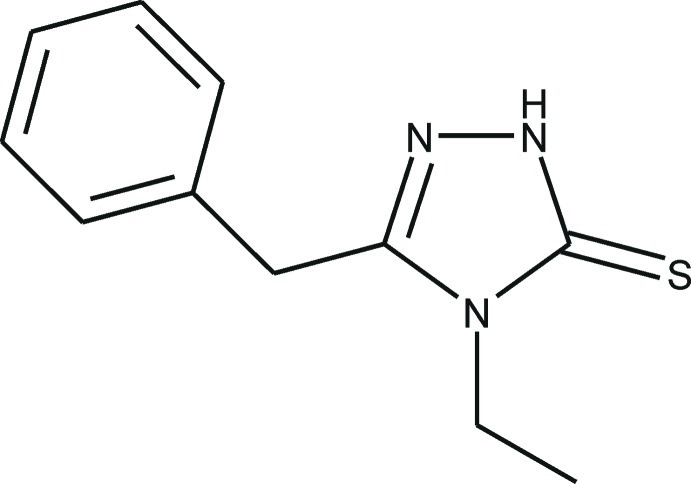


## Experimental

### 

#### Crystal data

C_11_H_13_N_3_S
*M* *_r_* = 219.30
Monoclinic, 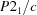

*a* = 7.3731 (5) Å
*b* = 8.9408 (19) Å
*c* = 16.9936 (8) Å
β = 91.892 (4)°
*V* = 1119.6 (3) Å^3^
*Z* = 4
Mo *K*α radiation
μ = 0.26 mm^−1^
*T* = 296 K
0.55 × 0.20 × 0.20 mm

#### Data collection

Kuma KM-4 four-circle diffractometer
Absorption correction: ψ scan (North *et al.*, 1968 ▶) *T*_min_ = 0.834, *T*_max_ = 0.852
3392 measured reflections
3289 independent reflections
1385 reflections with *I* > 2σ(*I*)
*R*_int_ = 0.039
2 standard reflections every 100 reflections intensity decay: 1%

#### Refinement

*R*[*F* ^2^ > 2σ(*F* ^2^)] = 0.047
*wR*(*F* ^2^) = 0.156
*S* = 0.98
3289 reflections
187 parameters
6 restraints
H atoms treated by a mixture of independent and constrained refinement
Δρ_max_ = 0.23 e Å^−3^
Δρ_min_ = −0.20 e Å^−3^

### 

Data collection: *KM4B8* (Gałdecki *et al.*, 1996 ▶); cell refinement: *KM4B8*; data reduction: *DATAPROC* (Gałdecki *et al.*, 1995 ▶); program(s) used to solve structure: *SHELXS97* (Sheldrick, 2008 ▶); program(s) used to refine structure: *SHELXL97* (Sheldrick, 2008 ▶); molecular graphics: *ORTEP-3 for Windows* (Farrugia, 2012 ▶); software used to prepare material for publication: *SHELXL97* and *WinGX* (Farrugia, 2012 ▶).

## Supplementary Material

Crystal structure: contains datablock(s) I, global. DOI: 10.1107/S1600536812051276/fy2077sup1.cif

Structure factors: contains datablock(s) I. DOI: 10.1107/S1600536812051276/fy2077Isup2.hkl

Supplementary material file. DOI: 10.1107/S1600536812051276/fy2077Isup3.cml

Additional supplementary materials:  crystallographic information; 3D view; checkCIF report

## Figures and Tables

**Table 1:** Hydrogen-bond geometry (Å, °)

*D*—H⋯*A*	*D*—H	H⋯*A*	*D*⋯*A*	*D*—H⋯*A*
C7—H7*B*⋯S6	0.97	2.85	3.204 (3)	103
N1—H1⋯S6^i^	0.86 (3)	2.46 (3)	3.303 (3)	167 (3)

Symmetry code: (i) 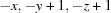.

**Fig. 1.**
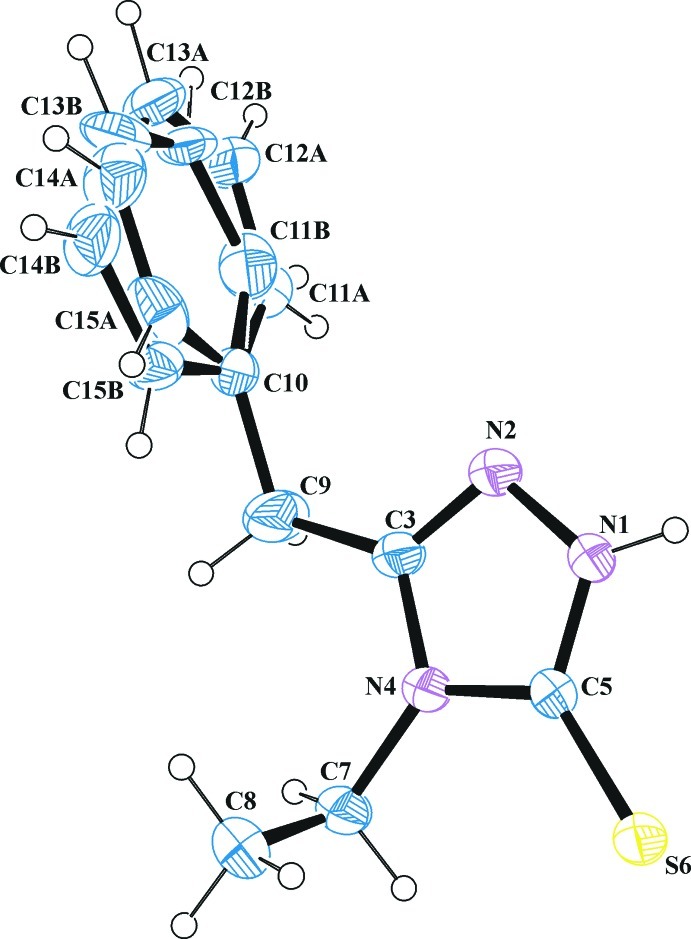
The molecular structure of (I), with atom labels and 30% probability displacement ellipsoids for non-H atoms.

**Fig. 2.**
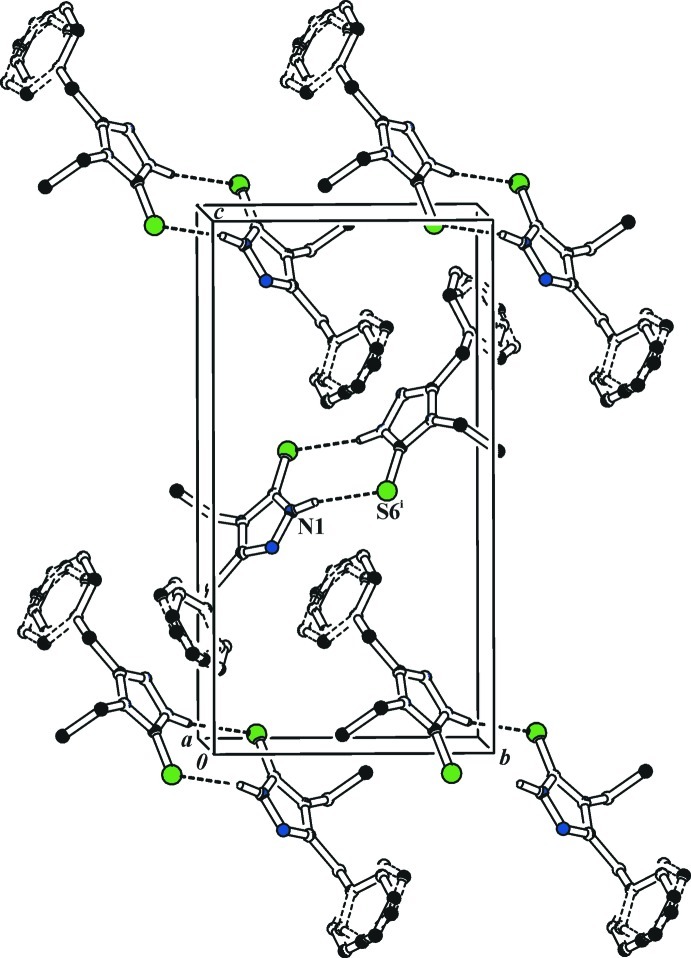
A view of the molecular packing in (I).

## supplementary crystallographic information

### Comment

The 1,2,4-triazole-5-thiones were found to have significant antimicrobial action (Saadeh *et al.*, 2010; Akhtar *et al.*, 2008; Al-Omar *et al*., 2010). The title compound, (I), belongs to 3- and 4-substituted derivatives of 1,2,4-triazole-5-thiones with potential antituberculosis activity against mycobacterium strains of *Mycobacterium smegmatis*, *Mycobacterium phlei* and *Mycobacterium H37Ra* (Pitucha *et al.*, 2010).

The X-ray analysis of the title compound revealed that this compound exists as the 5-thioxo tautomer in the crystalline state. The molecular geometry of (I) is very similar to that observed in the related structures of ethyl 2-(3-methyl-5-sulfanylidene-4,5-dihydro-1*H*-1,2,4-triazol-4-yl)acetate (Karczmarzyk *et al.*, 2012), 2-(3-methyl-5-thioxo-4,5-dihydro-1*H*-1,2,4-triazol-4-yl)acetic acid (Kruszynski *et al.*, 2007) and 4-[3-(2-methyl-furan-3-yl)-5-thioxo-1,2,4-triazol-4-yl]acetic acid (Siwek *et al.*, 2008). The 1,2,4-triazole ring is planar to within 0.002 (3) Å. The benzene ring exhibits disorder giving two components A and B with a common bridgehead C atom. The benzyl group adopts a *cis*-*gauche* conformation in respect to 1,2,4-triazole ring with the torsion angles N2—C3—C9—C10 and C3—C9—C10—C11A for benzene ring A and C3—C9—C10—C11B for benzene ring B of 25.1 (5), -102.9 (9) and -105 (4)°, respectively. The plane of the ethyl chain is positioned almost perpendicular to the mean plane of the 1,2,4-triazole ring with the dihedral angle of 88.55 (15)°. This conformation is stabilized by the C7—H71···S6 intramolecular hydrogen bond specified as S(5) in graph set notation (Bernstein *et al.*, 1995).

In the crystal structure (Fig. 2), inversion-related molecules of (I) form molecular dimers designated as R^2^_2_(8) rings *via* N1—H1···S6 intermolecular hydrogen bonds. Moreover, the *π*-electron systems of the pairs of triazole and benzene rings belonging to the molecules related by 2_1_ axis overlap each other, with centroid-to-centroid separation of 3.547 (4) Å for ring A and 3.544 (12) Å for ring B between the triazole ring at (*x*, *y*, *z*) and benzene rings at (-*x*, *y*+1/2, -*z*+1/2) and benzene rings at (*x*, *y*, *z*) and triazole ring at (-*x*, *y*-1/2, -*z*+1/2). The angle between overlapping planes is 6.6 (3)° for A and 5.0 (10)° for B and the slippage is 0.490 (58) and 0.575 (147) Å for rings A and B, respectively.

### Experimental

The title compound, (I), was prepared by the cyclization reaction of 1-benzyl-4-ethylthiosemicarbazide in alkaline medium according to the metod described by Dobosz & Pachuta-Stec (1996). Crystals uitable for X-ray diffraction analysis were grown by slow evaporation of ethanol solution.

### Refinement

The benzene ring exhibits disorder with the refined ratio of 0.77 (2):0.23 (2) for the two components A and B with common bridgehead C atom. DFIX restraints (SHELXL97; Sheldrick, 2008) with a target value of 1.380 (5) Å were used for all C≐C bonds in component B. The N-bound H atom was located by difference Fourier synthesis and refined freely. The remaining H atoms were positioned geometrically and treated as riding on their C atoms with C—H distances of 0.93 Å (aromatic), 0.96 Å (CH_2_) and 0.97 Å (CH_3_). All H atoms were assigned *U*_iso_(H) values of 1.5*U*_eq_(N,C).

### Crystal data

**Table:**

C_11_H_13_N_3_S	*F*(000) = 464
*M**_r_* = 219.30	*D*_x_ = 1.301 Mg m^−^^3^
Monoclinic, *P*2_1_/*c*	Melting point: 425 K
Hall symbol: -P 2ybc	Mo *K*α radiation, λ = 0.71073 Å
*a* = 7.3731 (5) Å	Cell parameters from 67 reflections
*b* = 8.9408 (19) Å	θ = 3.6–11.2°
*c* = 16.9936 (8) Å	µ = 0.26 mm^−^^1^
β = 91.892 (4)°	*T* = 296 K
*V* = 1119.6 (3) Å^3^	Prism, colourless
*Z* = 4	0.55 × 0.20 × 0.20 mm

### Data collection

**Table:**

Kuma KM-4 four-circle diffractometer	1385 reflections with *I* > 2σ(*I*)
Radiation source: fine-focus sealed tube	*R*_int_ = 0.039
Graphite monochromator	θ_max_ = 30.1°, θ_min_ = 2.4°
ω–2θ scans	*h* = −10→10
Absorption correction: ψ scan (North *et al.*, 1968)	*k* = 0→12
*T*_min_ = 0.834, *T*_max_ = 0.852	*l* = 0→23
3392 measured reflections	2 standard reflections every 100 reflections
3289 independent reflections	intensity decay: 1%

### Refinement

**Table:**

Refinement on *F*^2^	Secondary atom site location: difference Fourier map
Least-squares matrix: full	Hydrogen site location: difference Fourier map
*R*[*F*^2^ > 2σ(*F*^2^)] = 0.047	H atoms treated by a mixture of independent and constrained refinement
*wR*(*F*^2^) = 0.156	*w* = 1/[σ^2^(*F*_o_^2^) + (0.062*P*)^2^ + 0.2203*P*] where *P* = (*F*_o_^2^ + 2*F*_c_^2^)/3
*S* = 0.98	(Δ/σ)_max_ < 0.001
3289 reflections	Δρ_max_ = 0.23 e Å^−^^3^
187 parameters	Δρ_min_ = −0.20 e Å^−^^3^
6 restraints	Extinction correction: *SHELXL97* (Sheldrick, 2008), Fc^*^=kFc[1+0.001xFc^2^λ^3^/sin(2θ)]^-1/4^
Primary atom site location: structure-invariant direct methods	Extinction coefficient: 0.012 (3)

### Special details

**Table:**

Geometry. All esds (except the esd in the dihedral angle between two l.s. planes) are estimated using the full covariance matrix. The cell esds are taken into account individually in the estimation of esds in distances, angles and torsion angles; correlations between esds in cell parameters are only used when they are defined by crystal symmetry. An approximate (isotropic) treatment of cell esds is used for estimating esds involving l.s. planes.
Refinement. Refinement of F^2^ against ALL reflections. The weighted R-factor wR and goodness of fit S are based on F^2^, conventional R-factors R are based on F, with F set to zero for negative F^2^. The threshold expression of F^2^ > 2sigma(F^2^) is used only for calculating R-factors(gt) etc. and is not relevant to the choice of reflections for refinement. R-factors based on F^2^ are statistically about twice as large as those based on F, and R- factors based on ALL data will be even larger.

### Fractional atomic coordinates and isotropic or equivalent isotropic displacement parameters (Å^2^)

**Table:**

	*x*	*y*	*z*	*U*_iso_*/*U*_eq_	Occ. (<1)
S6	−0.22230 (10)	0.33538 (8)	0.53097 (4)	0.0567 (3)	
N1	0.0496 (3)	0.3323 (3)	0.42647 (13)	0.0473 (6)	
H1	0.101 (4)	0.412 (4)	0.4446 (18)	0.071*	
N2	0.1063 (3)	0.2595 (3)	0.36067 (13)	0.0525 (6)	
N4	−0.1519 (3)	0.1693 (2)	0.40086 (12)	0.0445 (5)	
C3	−0.0184 (4)	0.1613 (3)	0.34683 (16)	0.0516 (7)	
C5	−0.1070 (3)	0.2807 (3)	0.45287 (15)	0.0412 (6)	
C7	−0.3151 (4)	0.0780 (3)	0.40482 (17)	0.0563 (8)	
H7A	−0.3527	0.0466	0.3521	0.084*	
H7B	−0.4122	0.1375	0.4259	0.084*	
C8	−0.2841 (5)	−0.0576 (4)	0.4557 (2)	0.0776 (11)	
H8A	−0.2561	−0.0268	0.5088	0.116*	
H8B	−0.1847	−0.1146	0.4362	0.116*	
H8C	−0.3917	−0.1182	0.4545	0.116*	
C9	−0.0260 (4)	0.0531 (4)	0.2795 (2)	0.0834 (12)	
H91	−0.1034	0.0942	0.2376	0.125*	
H92	−0.0813	−0.0391	0.2969	0.125*	
C10	0.1561 (3)	0.0173 (3)	0.24685 (16)	0.0456 (6)	
C11A	0.1986 (18)	0.0849 (15)	0.1776 (6)	0.0499 (18)	0.77 (2)
H11A	0.1175	0.1531	0.1547	0.075*	0.77 (2)
C12A	0.3582 (15)	0.0546 (11)	0.1410 (6)	0.062 (2)	0.77 (2)
H12A	0.3837	0.1027	0.0941	0.093*	0.77 (2)
C13A	0.4806 (9)	−0.0468 (11)	0.1733 (7)	0.066 (2)	0.77 (2)
H13A	0.5890	−0.0673	0.1490	0.100*	0.77 (2)
C14A	0.4388 (12)	−0.1159 (11)	0.2417 (7)	0.071 (3)	0.77 (2)
H14A	0.5179	−0.1870	0.2633	0.107*	0.77 (2)
C15A	0.2833 (14)	−0.0828 (10)	0.2790 (5)	0.068 (2)	0.77 (2)
H15A	0.2613	−0.1279	0.3270	0.102*	0.77 (2)
C11B	0.240 (5)	0.060 (6)	0.179 (2)	0.069 (11)	0.23 (2)
H11B	0.1873	0.1316	0.1463	0.103*	0.23 (2)
C12B	0.403 (3)	−0.004 (4)	0.1598 (16)	0.052 (8)	0.23 (2)
H12B	0.4608	0.0185	0.1134	0.077*	0.23 (2)
C13B	0.475 (3)	−0.104 (4)	0.214 (2)	0.074 (12)	0.23 (2)
H13B	0.5909	−0.1414	0.2053	0.112*	0.23 (2)
C14B	0.391 (3)	−0.154 (3)	0.280 (2)	0.072 (7)	0.23 (2)
H14B	0.4437	−0.2272	0.3126	0.108*	0.23 (2)
C15B	0.225 (3)	−0.093 (3)	0.2963 (16)	0.053 (6)	0.23 (2)
H15B	0.1608	−0.1244	0.3395	0.079*	0.23 (2)

### Atomic displacement parameters (Å^2^)

**Table:**

	*U*^11^	*U*^22^	*U*^33^	*U*^12^	*U*^13^	*U*^23^
S6	0.0669 (5)	0.0494 (4)	0.0553 (4)	−0.0142 (4)	0.0264 (3)	−0.0107 (4)
N1	0.0485 (13)	0.0404 (12)	0.0538 (13)	−0.0110 (11)	0.0150 (10)	−0.0100 (11)
N2	0.0480 (13)	0.0527 (13)	0.0577 (15)	−0.0092 (11)	0.0180 (11)	−0.0129 (12)
N4	0.0407 (11)	0.0443 (12)	0.0491 (12)	−0.0089 (10)	0.0113 (9)	−0.0094 (11)
C3	0.0438 (14)	0.0560 (16)	0.0558 (16)	−0.0092 (14)	0.0160 (12)	−0.0166 (14)
C5	0.0429 (14)	0.0345 (12)	0.0465 (15)	−0.0038 (11)	0.0069 (11)	0.0005 (11)
C7	0.0447 (15)	0.0585 (18)	0.0665 (19)	−0.0148 (14)	0.0138 (13)	−0.0140 (15)
C8	0.080 (2)	0.0522 (18)	0.102 (3)	−0.0176 (17)	0.031 (2)	−0.0055 (18)
C9	0.0629 (19)	0.101 (3)	0.089 (2)	−0.0244 (19)	0.0288 (17)	−0.054 (2)
C10	0.0451 (15)	0.0458 (15)	0.0465 (15)	−0.0063 (12)	0.0078 (12)	−0.0113 (13)
C11A	0.052 (4)	0.043 (4)	0.055 (4)	0.004 (3)	0.006 (3)	−0.004 (2)
C12A	0.062 (5)	0.063 (5)	0.062 (4)	0.001 (3)	0.024 (3)	−0.004 (3)
C13A	0.043 (4)	0.077 (5)	0.080 (5)	0.008 (3)	0.014 (4)	−0.027 (4)
C14A	0.063 (6)	0.063 (5)	0.087 (8)	0.018 (4)	−0.015 (5)	0.002 (4)
C15A	0.091 (6)	0.063 (4)	0.050 (4)	−0.020 (5)	−0.003 (4)	0.007 (3)
C11B	0.054 (19)	0.06 (2)	0.09 (2)	0.006 (12)	−0.022 (13)	−0.003 (13)
C12B	0.032 (14)	0.07 (2)	0.052 (16)	−0.003 (12)	0.015 (12)	−0.019 (14)
C13B	0.051 (12)	0.10 (2)	0.07 (2)	−0.041 (13)	0.029 (13)	−0.041 (18)
C14B	0.074 (15)	0.049 (11)	0.094 (19)	0.016 (10)	−0.004 (13)	0.003 (11)
C15B	0.033 (10)	0.059 (12)	0.067 (15)	0.008 (9)	0.003 (7)	0.008 (8)

### Geometric parameters (Å, º)

**Table:**

S6—C5	1.673 (3)	C10—C11B	1.379 (5)
N1—C5	1.335 (3)	C10—C15A	1.394 (7)
N1—N2	1.371 (3)	C11A—C12A	1.376 (9)
N1—H1	0.86 (3)	C11A—H11A	0.9300
N2—C3	1.288 (3)	C12A—C13A	1.381 (10)
N4—C5	1.365 (3)	C12A—H12A	0.9300
N4—C3	1.370 (3)	C13A—C14A	1.360 (11)
N4—C7	1.457 (3)	C13A—H13A	0.9300
C3—C9	1.498 (4)	C14A—C15A	1.361 (9)
C7—C8	1.502 (4)	C14A—H14A	0.9300
C7—H7A	0.9700	C15A—H15A	0.9300
C7—H7B	0.9700	C11B—C12B	1.380 (5)
C8—H8A	0.9600	C11B—H11B	0.9300
C8—H8B	0.9600	C12B—C13B	1.379 (5)
C8—H8C	0.9600	C12B—H12B	0.9300
C9—C10	1.504 (4)	C13B—C14B	1.377 (5)
C9—H91	0.9700	C13B—H13B	0.9300
C9—H92	0.9700	C14B—C15B	1.379 (5)
C10—C11A	1.369 (6)	C14B—H14B	0.9300
C10—C15B	1.379 (5)	C15B—H15B	0.9300
			
C5—N1—N2	113.6 (2)	C15B—C10—C9	103.9 (10)
C5—N1—H1	123 (2)	C11B—C10—C9	133.0 (12)
N2—N1—H1	123 (2)	C15A—C10—C9	126.1 (5)
C3—N2—N1	103.7 (2)	C10—C11A—C12A	121.7 (8)
C5—N4—C3	107.9 (2)	C10—C11A—H11A	119.2
C5—N4—C7	124.2 (2)	C12A—C11A—H11A	119.2
C3—N4—C7	127.9 (2)	C11A—C12A—C13A	120.5 (9)
N2—C3—N4	111.5 (2)	C11A—C12A—H12A	119.8
N2—C3—C9	126.0 (2)	C13A—C12A—H12A	119.8
N4—C3—C9	122.4 (2)	C14A—C13A—C12A	118.3 (8)
N1—C5—N4	103.2 (2)	C14A—C13A—H13A	120.8
N1—C5—S6	129.3 (2)	C12A—C13A—H13A	120.8
N4—C5—S6	127.47 (18)	C13A—C14A—C15A	121.1 (7)
N4—C7—C8	111.6 (2)	C13A—C14A—H14A	119.4
N4—C7—H7A	109.3	C15A—C14A—H14A	119.4
C8—C7—H7A	109.3	C14A—C15A—C10	121.6 (5)
N4—C7—H7B	109.3	C14A—C15A—H15A	119.2
C8—C7—H7B	109.3	C10—C15A—H15A	119.2
H7A—C7—H7B	108.0	C10—C11B—C12B	120.3 (17)
C7—C8—H8A	109.5	C10—C11B—H11B	119.8
C7—C8—H8B	109.5	C12B—C11B—H11B	119.8
H8A—C8—H8B	109.5	C13B—C12B—C11B	115 (2)
C7—C8—H8C	109.5	C13B—C12B—H12B	122.3
H8A—C8—H8C	109.5	C11B—C12B—H12B	122.3
H8B—C8—H8C	109.5	C14B—C13B—C12B	126 (2)
C3—C9—C10	114.1 (3)	C14B—C13B—H13B	117.2
C3—C9—H91	108.7	C12B—C13B—H13B	117.2
C10—C9—H91	108.7	C13B—C14B—C15B	117 (2)
C3—C9—H92	108.7	C13B—C14B—H14B	121.3
C10—C9—H92	108.7	C15B—C14B—H14B	121.3
H91—C9—H92	107.6	C14B—C15B—C10	118.3 (15)
C15B—C10—C11B	122.5 (12)	C14B—C15B—H15B	120.8
C11A—C10—C15A	116.8 (5)	C10—C15B—H15B	120.8
C11A—C10—C9	117.1 (5)		
			
C5—N1—N2—C3	−0.1 (3)	C3—C9—C10—C11B	−105 (4)
N1—N2—C3—N4	0.3 (3)	C3—C9—C10—C15A	79.6 (7)
N1—N2—C3—C9	178.3 (3)	C15A—C10—C11A—C12A	0.7 (18)
C5—N4—C3—N2	−0.3 (3)	C9—C10—C11A—C12A	−177.0 (11)
C7—N4—C3—N2	179.7 (3)	C10—C11A—C12A—C13A	0 (2)
C5—N4—C3—C9	−178.5 (3)	C11A—C12A—C13A—C14A	0.4 (17)
C7—N4—C3—C9	1.6 (5)	C12A—C13A—C14A—C15A	−2.2 (16)
N2—N1—C5—N4	−0.1 (3)	C13A—C14A—C15A—C10	3.3 (16)
N2—N1—C5—S6	179.3 (2)	C11A—C10—C15A—C14A	−2.5 (14)
C3—N4—C5—N1	0.2 (3)	C9—C10—C15A—C14A	175.0 (7)
C7—N4—C5—N1	−179.8 (2)	C15B—C10—C11B—C12B	−2 (7)
C3—N4—C5—S6	−179.2 (2)	C9—C10—C11B—C12B	−173 (3)
C7—N4—C5—S6	0.8 (4)	C10—C11B—C12B—C13B	−3 (7)
C5—N4—C7—C8	−88.5 (3)	C11B—C12B—C13B—C14B	7 (5)
C3—N4—C7—C8	91.4 (3)	C12B—C13B—C14B—C15B	−4 (5)
N2—C3—C9—C10	25.1 (5)	C13B—C14B—C15B—C10	−2 (5)
N4—C3—C9—C10	−157.0 (3)	C11B—C10—C15B—C14B	5 (5)
C3—C9—C10—C11A	−102.9 (9)	C9—C10—C15B—C14B	178 (3)
C3—C9—C10—C15B	83.1 (16)		

### Hydrogen-bond geometry (Å, º)

**Table:**

*D*—H···*A*	*D*—H	H···*A*	*D*···*A*	*D*—H···*A*
C7—H7*B*···S6	0.97	2.85	3.204 (3)	103
N1—H1···S6^i^	0.86 (3)	2.46 (3)	3.303 (3)	167 (3)

Symmetry code: (i) −*x*, −*y*+1, −*z*+1.
